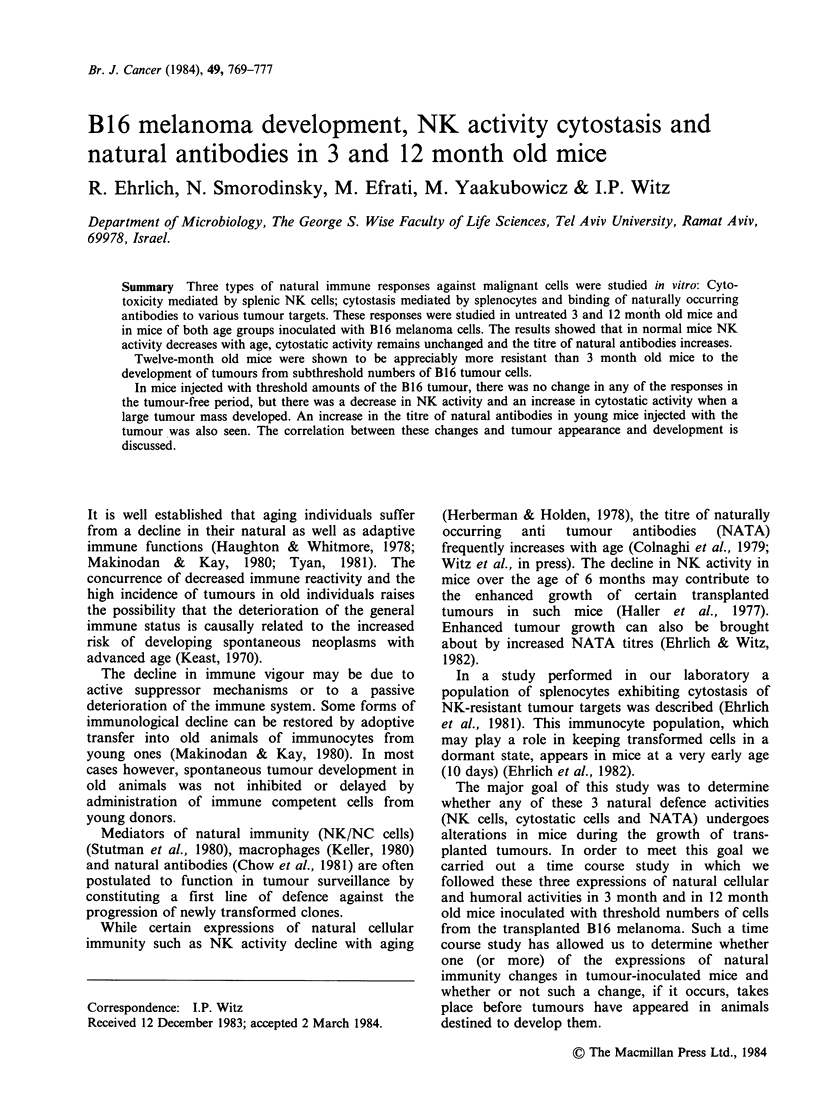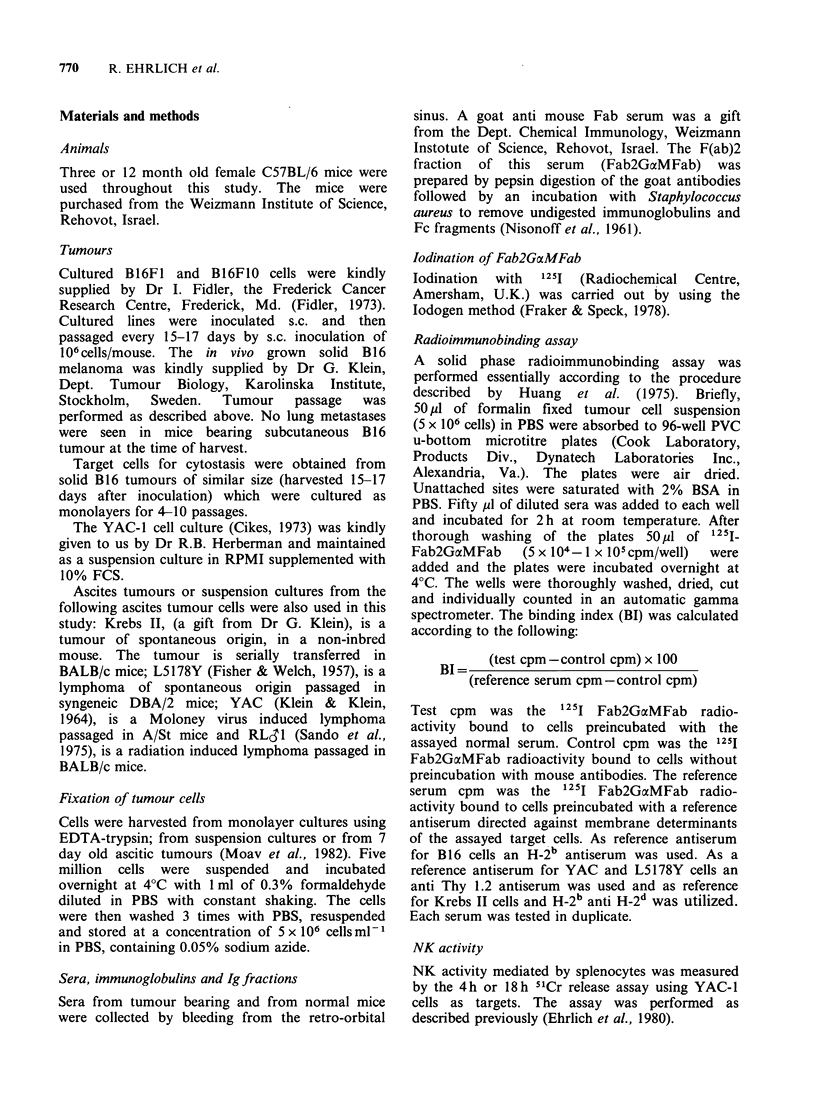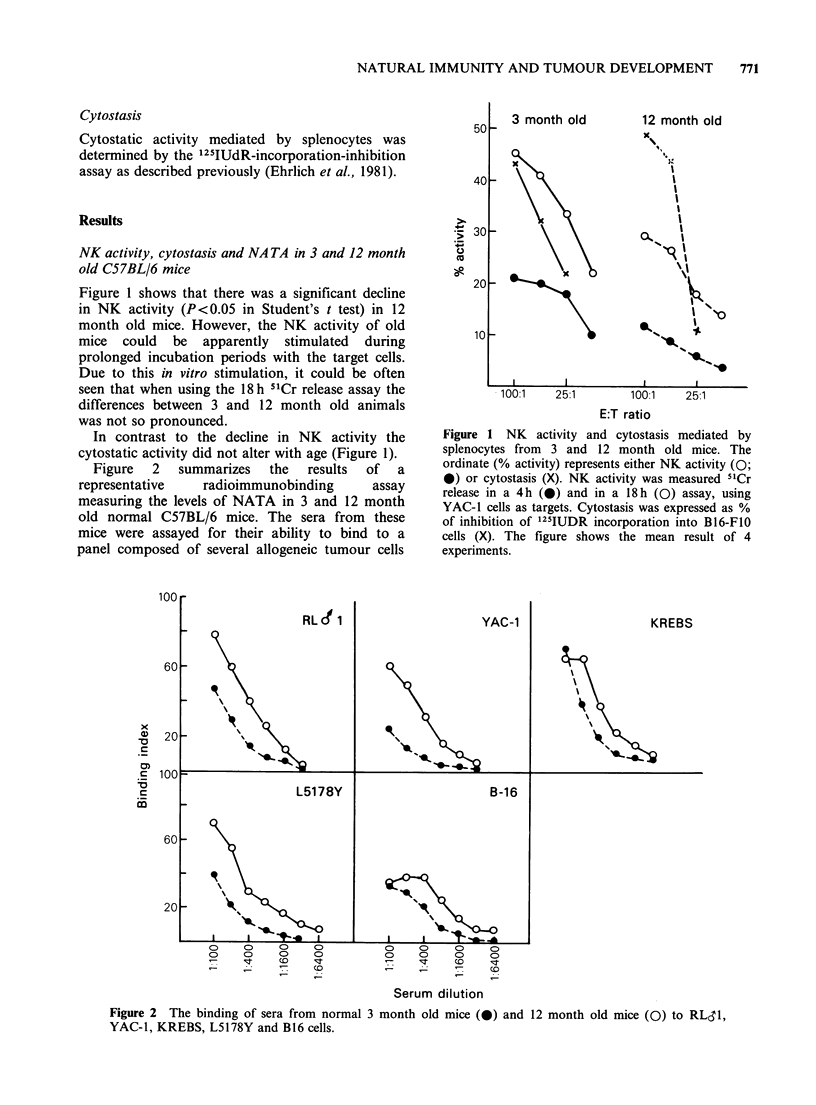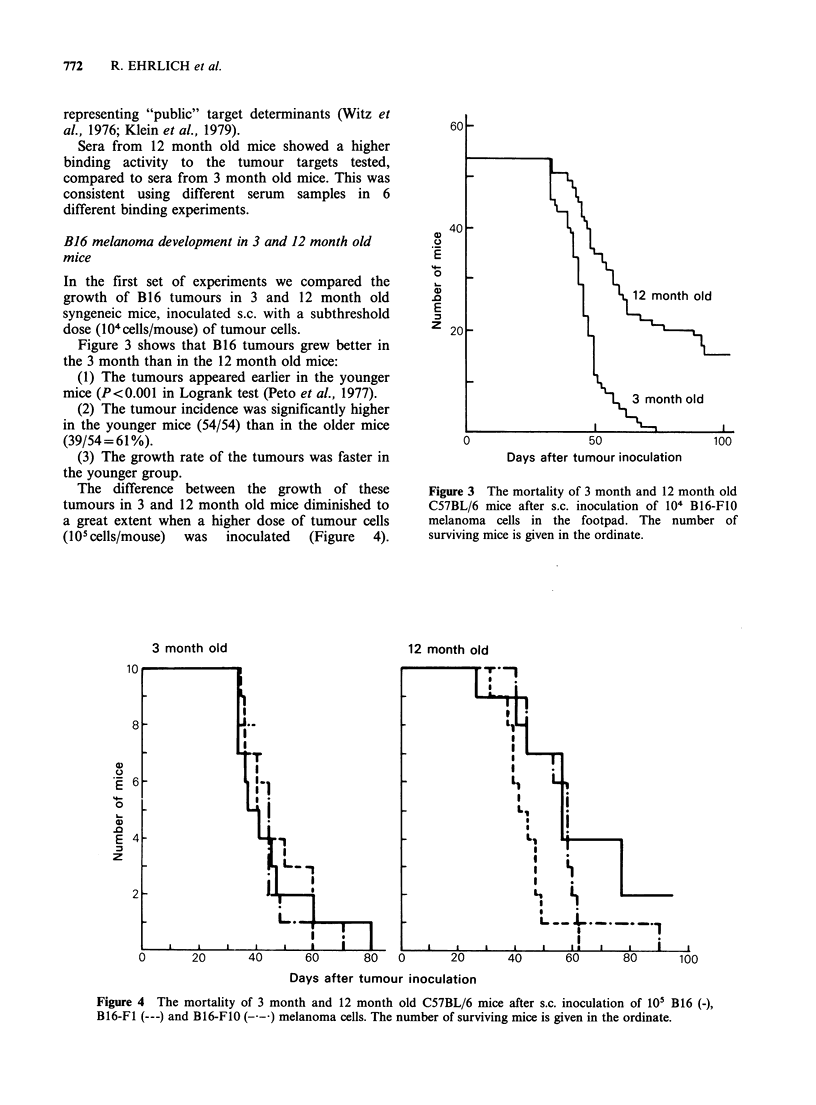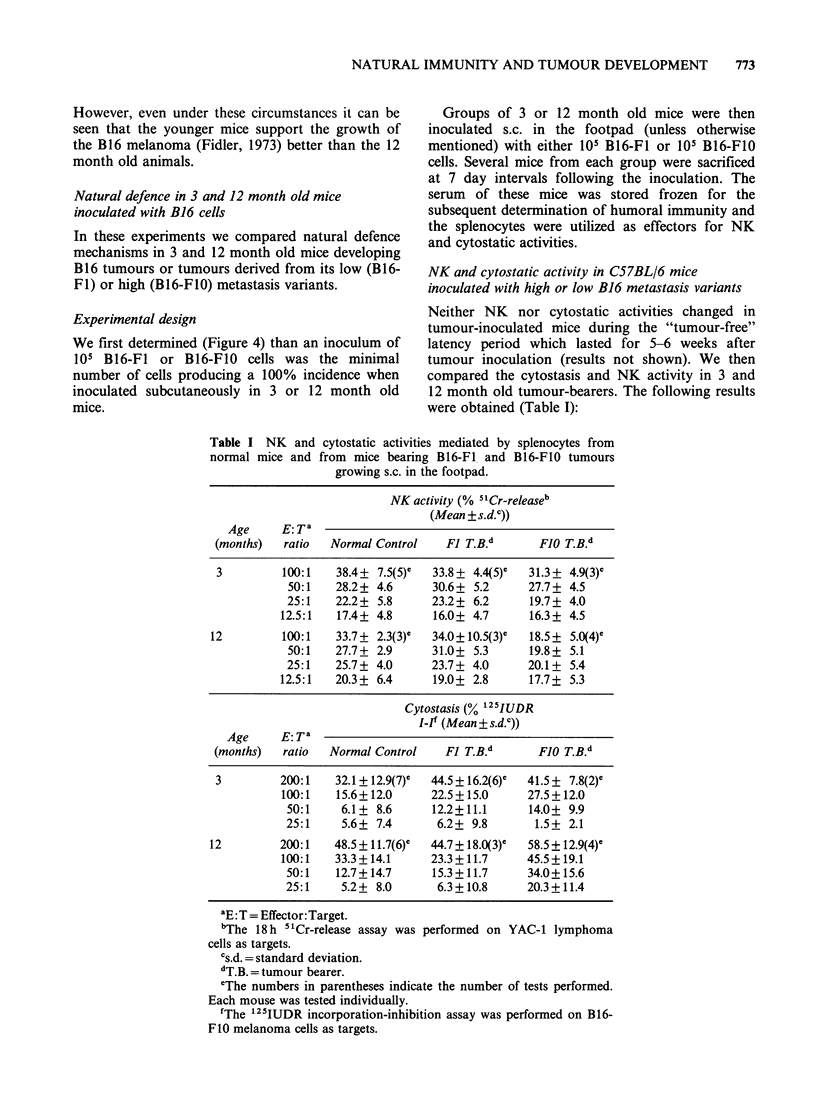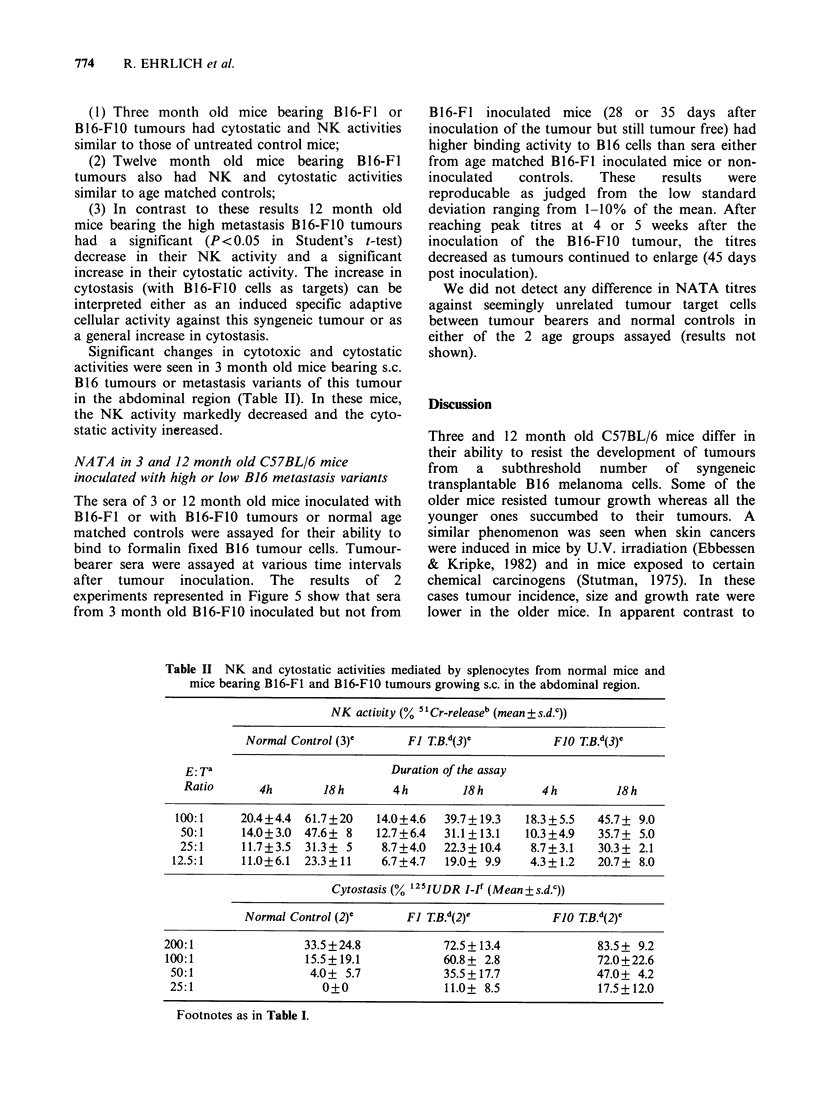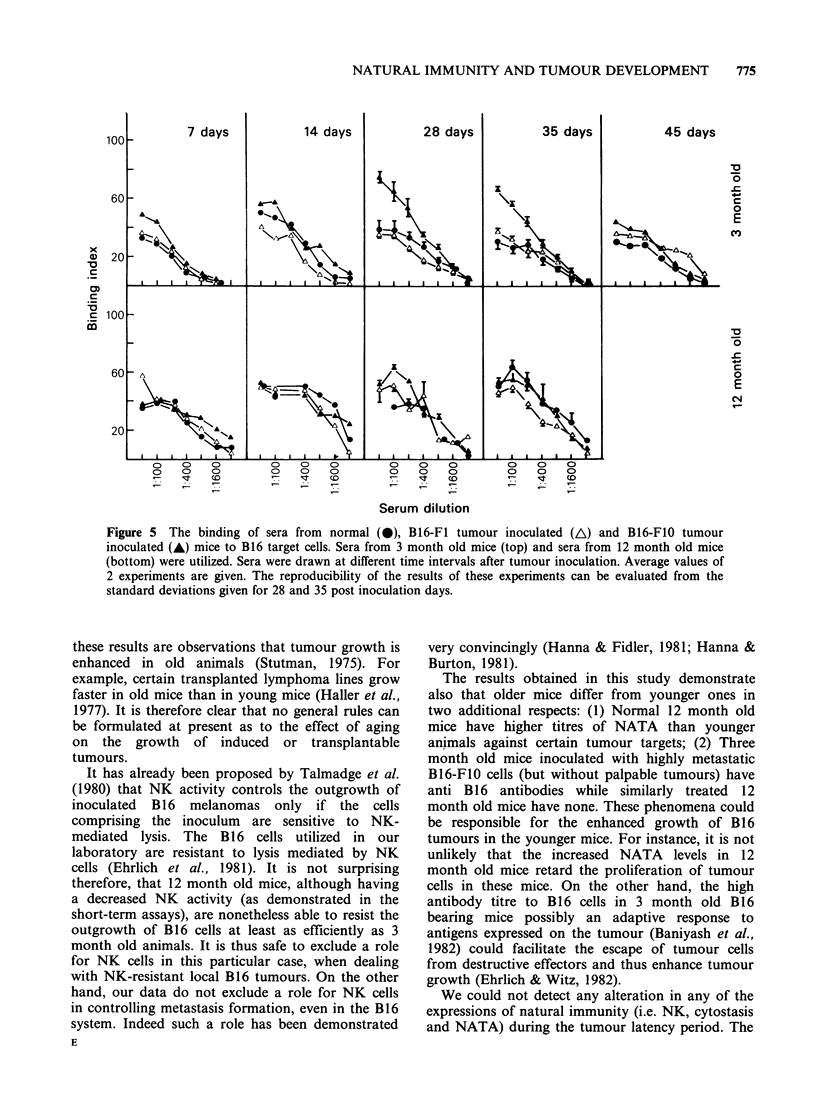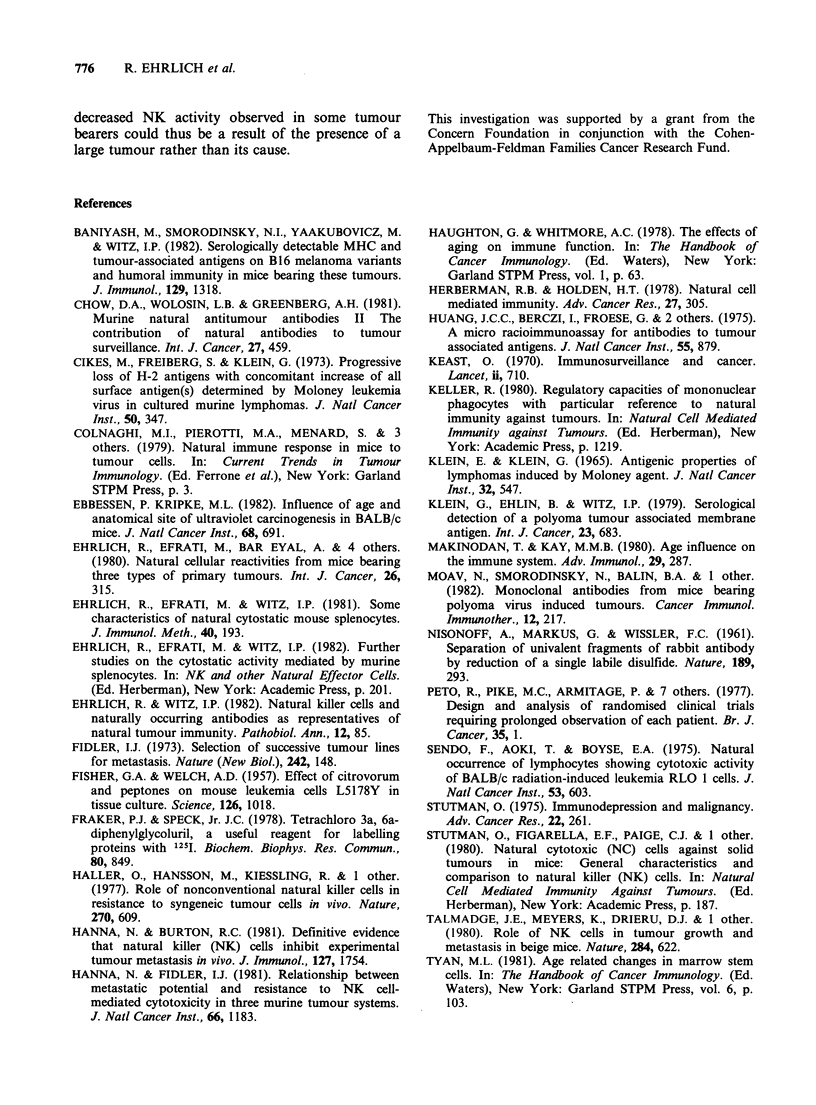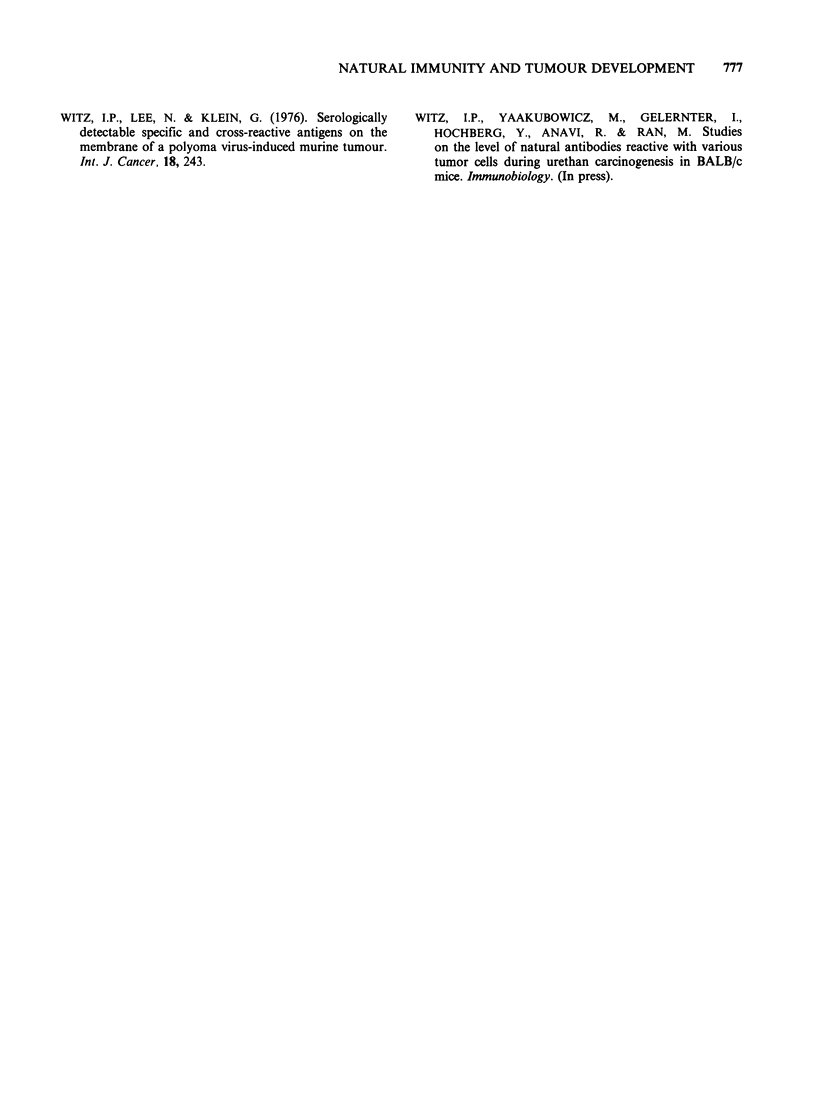# B16 melanoma development, NK activity cytostasis and natural antibodies in 3 and 12 month old mice.

**DOI:** 10.1038/bjc.1984.120

**Published:** 1984-06

**Authors:** R. Ehrlich, N. Smorodinsky, M. Efrati, M. Yaakubowicz, I. P. Witz

## Abstract

Three types of natural immune responses against malignant cells were studied in vitro: Cytotoxicity mediated by splenic NK cells; cytostasis mediated by splenocytes and binding of naturally occurring antibodies to various tumour targets. These responses were studied in untreated 3 and 12 month old mice and in mice of both age groups inoculated with B16 melanoma cells. The results showed that in normal mice NK activity decreases with age, cytostatic activity remains unchanged and the titre of natural antibodies increases. Twelve-month old mice were shown to be appreciably more resistant than 3 month old mice to the development of tumours from subthreshold numbers of B16 tumour cells. In mice injected with threshold amounts of the B16 tumour, there was no change in any of the responses in the tumour-free period, but there was a decrease in NK activity and an increase in cytostatic activity when a large tumour mass developed. An increase in the titre of natural antibodies in young mice injected with the tumour was also seen. The correlation between these changes and tumour appearance and development is discussed.


*Br. J. Cancer* (1984), **49**, 769–777

### B16 melanoma development, NK activity cytostasis and natural antibodies in 3 and 12 month old mice

R. Ehrlich, N. Smorodinsky, M. Efrati, M. Yaakubowicz & I.P. Witz

*Department of Microbiology, The George S. Wise Faculty of Life Sciences, Tel Aviv University, Ramat Aviv, 69978, Israel.*

**Summary** Three types of natural immune responses against malignant cells were studied *in vitro*: Cytotoxicity mediated by splenic NK cells; cytostasis mediated by splenocytes and binding of naturally occurring antibodies to various tumour targets. These responses were studied in untreated 3 and 12 month old mice and in mice of both age groups inoculated with B16 melanoma cells. The results showed that in normal mice NK activity decreases with age, cytostatic activity remains unchanged and the titre of natural antibodies increases.

Twelve-month old mice were shown to be appreciably more resistant than 3 month old mice to the development of tumours from subthreshold numbers of B16 tumour cells.

In mice injected with threshold amounts of the B16 tumour, there was no change in any of the responses in the tumour-free period, but there was a decrease in NK activity and an increase in cytostatic activity when a large tumour mass developed. An increase in the titre of natural antibodies in young mice injected with the tumour was also seen. The correlation between these changes and tumour appearance and development is discussed.

It is well established that aging individuals suffer from a decline in their natural as well as adaptive immune functions (Haughton & Whitmore, 1978; Makinodan & Kay, 1980; Tyan, 1981). The concurrence of decreased immune reactivity and the high incidence of tumours in old individuals raises the possibility that the deterioration of the general immune status is causally related to the increased risk of developing spontaneous neoplasms with advanced age (Keast, 1970).

The decline in immune vigour may be due to active suppressor mechanisms or to a passive deterioration of the immune system. Some forms of immunological decline can be restored by adoptive transfer into old animals of immunocytes from young ones (Makinodan & Kay, 1980). In most cases however, spontaneous tumour development in old animals was not inhibited or delayed by administration of immune competent cells from young donors.

Mediators of natural immunity (NK/NC cells) (Stutman *et al.*, 1980), macrophages (Keller, 1980) and natural antibodies (Chow *et al.*, 1981) are often postulated to function in tumour surveillance by constituting a first line of defence against the progression of newly transformed clones.

While certain expressions of natural cellular immunity such as NK activity decline with aging (Herberman & Holden, 1978), the titre of naturally occurring anti tumour antibodies (NATA) frequently increases with age (Colnaghi *et al.*, 1979; Witz *et al.*, in press). The decline in NK activity in mice over the age of 6 months may contribute to the enhanced growth of certain transplanted tumours in such mice (Haller *et al.*, 1977). Enhanced tumour growth can also be brought about by increased NATA titres (Ehrlich & Witz, 1982).

In a study performed in our laboratory a population of splenocytes exhibiting cytostasis of NK-resistant tumour targets was described (Ehrlich *et al.*, 1981). This immunocyte population, which may play a role in keeping transformed cells in a dormant state, appears in mice at a very early age (10 days) (Ehrlich *et al.*, 1982).

The major goal of this study was to determine whether any of these 3 natural defence activities (NK cells, cytostatic cells and NATA) undergoes alterations in mice during the growth of transplanted tumours. In order to meet this goal we carried out a time course study in which we followed these three expressions of natural cellular and humoral activities in 3 month and in 12 month old mice inoculated with threshold numbers of cells from the transplanted B16 melanoma. Such a time course study has allowed us to determine whether one (or more) of the expressions of natural immunity changes in tumour-inoculated mice and whether or not such a change, if it occurs, takes place before tumours have appeared in animals destined to develop them.

Correspondence: I.P. Witz
Received 12 December 1983; accepted 2 March 1984.

© The Macmillan Press Ltd., 1984

### Materials and methods

*Animals*

Three or 12 month old female C57BL/6 mice were used throughout this study. The mice were purchased from the Weizmann Institute of Science, Rehovot, Israel.

*Tumours*

Cultured B16F1 and B16F10 cells were kindly supplied by Dr I. Fidler, the Frederick Cancer Research Centre, Frederick, Md. (Fidler, 1973). Cultured lines were inoculated s.c. and then passaged every 15–17 days by s.c. inoculation of $10^6$ cells/mouse. The *in vivo* grown solid B16 melanoma was kindly supplied by Dr G. Klein, Dept. Tumour Biology, Karolinska Institute, Stockholm, Sweden. Tumour passage was performed as described above. No lung metastases were seen in mice bearing subcutaneous B16 tumour at the time of harvest.

Target cells for cytostasis were obtained from solid B16 tumours of similar size (harvested 15–17 days after inoculation) which were cultured as monolayers for 4–10 passages.

The YAC-1 cell culture (Cikes, 1973) was kindly given to us by Dr R.B. Herberman and maintained as a suspension culture in RPMI supplemented with 10% FCS.

Ascites tumours or suspension cultures from the following ascites tumour cells were also used in this study: Krebs II, (a gift from Dr G. Klein), is a tumour of spontaneous origin, in a non-inbred mouse. The tumour is serially transferred in BALB/c mice; L5178Y (Fisher & Welch, 1957), is a lymphoma of spontaneous origin passaged in syngeneic DBA/2 mice; YAC (Klein & Klein, 1964), is a Moloney virus induced lymphoma passaged in A/St mice and RL♂1 (Sando *et al.*, 1975), is a radiation induced lymphoma passaged in BALB/c mice.

*Fixation of tumour cells*

Cells were harvested from monolayer cultures using EDTA-trypsin; from suspension cultures or from 7 day old ascitic tumours (Moav *et al.*, 1982). Five million cells were suspended and incubated overnight at 4°C with 1 ml of 0.3% formaldehyde diluted in PBS with constant shaking. The cells were then washed 3 times with PBS, resuspended and stored at a concentration of $5 \times 10^6$ cells ml$^{-1}$ in PBS, containing 0.05% sodium azide.

*Sera, immunoglobulins and Ig fractions*

Sera from tumour bearing and from normal mice were collected by bleeding from the retro-orbital sinus. A goat anti mouse Fab serum was a gift from the Dept. Chemical Immunology, Weizmann Instotute of Science, Rehovot, Israel. The F(ab)2 fraction of this serum (Fab2GαMFab) was prepared by pepsin digestion of the goat antibodies followed by an incubation with *Staphylococcus aureus* to remove undigested immunoglobulins and Fc fragments (Nisonoff *et al.*, 1961).

*Iodination of Fab2GαMFab*

Iodination with $^{125}$I (Radiochemical Centre, Amersham, U.K.) was carried out by using the Iodogen method (Fraker & Speck, 1978).

*Radioimmunobinding assay*

A solid phase radioimmunobinding assay was performed essentially according to the procedure described by Huang *et al.* (1975). Briefly, 50 μl of formalin fixed tumour cell suspension ($5 \times 10^6$ cells) in PBS were absorbed to 96-well PVC u-bottom microtitre plates (Cook Laboratory, Products Div., Dynatech Laboratories Inc., Alexandria, Va.). The plates were air dried. Unattached sites were saturated with 2% BSA in PBS. Fifty μl of diluted sera was added to each well and incubated for 2 h at room temperature. After thorough washing of the plates 50 μl of $^{125}$I-Fab2GαMFab ($5 \times 10^4 - 1 \times 10^5$ cpm/well) were added and the plates were incubated overnight at 4°C. The wells were thoroughly washed, dried, cut and individually counted in an automatic gamma spectrometer. The binding index (BI) was calculated according to the following:

$$BI = \frac{(\text{test cpm} - \text{control cpm}) \times 100}{(\text{reference serum cpm} - \text{control cpm})}$$

Test cpm was the $^{125}$I Fab2GαMFab radioactivity bound to cells preincubated with the assayed normal serum. Control cpm was the $^{125}$I Fab2GαMFab radioactivity bound to cells without preincubation with mouse antibodies. The reference serum cpm was the $^{125}$I Fab2GαMFab radioactivity bound to cells preincubated with a reference antiserum directed against membrane determinants of the assayed target cells. As reference antiserum for B16 cells an H-$2^b$ antiserum was used. As a reference antiserum for YAC and L5178Y cells an anti Thy 1.2 antiserum was used and as reference for Krebs II cells and H-$2^b$ anti H-$2^d$ was utilized. Each serum was tested in duplicate.

*NK activity*

NK activity mediated by splenocytes was measured by the 4 h or 18 h $^{51}$Cr release assay using YAC-1 cells as targets. The assay was performed as described previously (Ehrlich *et al.*, 1980).

*Cytostasis*

Cytostatic activity mediated by splenocytes was determined by the $^{125}$IUdR-incorporation-inhibition assay as described previously (Ehrlich *et al.*, 1981).

### Results

*NK activity, cytostasis and NATA in 3 and 12 month old C57BL/6 mice*

Figure 1 shows that there was a significant decline in NK activity ($P<0.05$ in Student's $t$ test) in 12 month old mice. However, the NK activity of old mice could be apparently stimulated during prolonged incubation periods with the target cells. Due to this *in vitro* stimulation, it could be often seen that when using the 18 h $^{51}$Cr release assay the differences between 3 and 12 month old animals was not so pronounced.

In contrast to the decline in NK activity the cytostatic activity did not alter with age (Figure 1).

Figure 2 summarizes the results of a representative radioimmunobinding assay measuring the levels of NATA in 3 and 12 month old normal C57BL/6 mice. The sera from these mice were assayed for their ability to bind to a panel composed of several allogeneic tumour cells

**Figure 1** NK activity and cytostasis mediated by splenocytes from 3 and 12 month old mice. The ordinate (% activity) represents either NK activity (○; ●) or cytostasis (X). NK activity was measured $^{51}$Cr release in a 4 h (●) and in a 18 h (○) assay, using YAC-1 cells as targets. Cytostasis was expressed as % of inhibition of $^{125}$IUDR incorporation into B16-F10 cells (X). The figure shows the mean result of 4 experiments.

**Figure 2** The binding of sera from normal 3 month old mice (●) and 12 month old mice (○) to RL♂1, YAC-1, KREBS, L5178Y and B16 cells.

representing "public" target determinants (Witz *et al.*, 1976; Klein *et al.*, 1979).

Sera from 12 month old mice showed a higher binding activity to the tumour targets tested, compared to sera from 3 month old mice. This was consistent using different serum samples in 6 different binding experiments.

*B16 melanoma development in 3 and 12 month old mice*

In the first set of experiments we compared the growth of B16 tumours in 3 and 12 month old syngeneic mice, inoculated s.c. with a subthreshold dose ($10^4$ cells/mouse) of tumour cells.

Figure 3 shows that B16 tumours grew better in the 3 month than in the 12 month old mice:

(1) The tumours appeared earlier in the younger mice ($P<0.001$ in Logrank test (Peto *et al.*, 1977).

(2) The tumour incidence was significantly higher in the younger mice (54/54) than in the older mice (39/54 = 61%).

(3) The growth rate of the tumours was faster in the younger group.

The difference between the growth of these tumours in 3 and 12 month old mice diminished to a great extent when a higher dose of tumour cells ($10^5$ cells/mouse) was inoculated (Figure 4).

**Figure 3** The mortality of 3 month and 12 month old C57BL/6 mice after s.c. inoculation of $10^4$ B16-F10 melanoma cells in the footpad. The number of surviving mice is given in the ordinate.

**Figure 4** The mortality of 3 month and 12 month old C57BL/6 mice after s.c. inoculation of $10^5$ B16 (-), B16-F1 (---) and B16-F10 (–·–·) melanoma cells. The number of surviving mice is given in the ordinate.

However, even under these circumstances it can be seen that the younger mice support the growth of the B16 melanoma (Fidler, 1973) better than the 12 month old animals.

*Natural defence in 3 and 12 month old mice inoculated with B16 cells*

In these experiments we compared natural defence mechanisms in 3 and 12 month old mice developing B16 tumours or tumours derived from its low (B16-F1) or high (B16-F10) metastasis variants.

*Experimental design*

We first determined (Figure 4) than an inoculum of $10^5$ B16-F1 or B16-F10 cells was the minimal number of cells producing a 100% incidence when inoculated subcutaneously in 3 or 12 month old mice.

Groups of 3 or 12 month old mice were then inoculated s.c. in the footpad (unless otherwise mentioned) with either $10^5$ B16-F1 or $10^5$ B16-F10 cells. Several mice from each group were sacrificed at 7 day intervals following the inoculation. The serum of these mice was stored frozen for the subsequent determination of humoral immunity and the splenocytes were utilized as effectors for NK and cytostatic activities.

*NK and cytostatic activity in C57BL/6 mice inoculated with high or low B16 metastasis variants*

Neither NK nor cytostatic activities changed in tumour-inoculated mice during the "tumour-free" latency period which lasted for 5–6 weeks after tumour inoculation (results not shown). We then compared the cytostasis and NK activity in 3 and 12 month old tumour-bearers. The following results were obtained (Table I):

**Table I** NK and cytostatic activities mediated by splenocytes from normal mice and from mice bearing B16-F1 and B16-F10 tumours growing s.c. in the footpad.

| *Age (months)* | *E:T[a] ratio* | *NK activity (% $^{51}$Cr-release[b] (Mean ± s.d.[c]))* | | |
|---|---|---|---|---|
| | | *Normal Control* | *F1 T.B.[d]* | *F10 T.B.[d]* |
| 3 | 100:1 | 38.4 ± 7.5(5)[e] | 33.8 ± 4.4(5)[e] | 31.3 ± 4.9(3)[e] |
| | 50:1 | 28.2 ± 4.6 | 30.6 ± 5.2 | 27.7 ± 4.5 |
| | 25:1 | 22.2 ± 5.8 | 23.2 ± 6.2 | 19.7 ± 4.0 |
| | 12.5:1 | 17.4 ± 4.8 | 16.0 ± 4.7 | 16.3 ± 4.5 |
| 12 | 100:1 | 33.7 ± 2.3(3)[e] | 34.0 ± 10.5(3)[e] | 18.5 ± 5.0(4)[e] |
| | 50:1 | 27.7 ± 2.9 | 31.0 ± 5.3 | 19.8 ± 5.1 |
| | 25:1 | 25.7 ± 4.0 | 23.7 ± 4.0 | 20.1 ± 5.4 |
| | 12.5:1 | 20.3 ± 6.4 | 19.0 ± 2.8 | 17.7 ± 5.3 |

| *Age (months)* | *E:T[a] ratio* | *Cytostasis (% $^{125}$IUDR I-I[f] (Mean ± s.d.[c]))* | | |
|---|---|---|---|---|
| | | *Normal Control* | *F1 T.B.[d]* | *F10 T.B.[d]* |
| 3 | 200:1 | 32.1 ± 12.9(7)[e] | 44.5 ± 16.2(6)[e] | 41.5 ± 7.8(2)[e] |
| | 100:1 | 15.6 ± 12.0 | 22.5 ± 15.0 | 27.5 ± 12.0 |
| | 50:1 | 6.1 ± 8.6 | 12.2 ± 11.1 | 14.0 ± 9.9 |
| | 25:1 | 5.6 ± 7.4 | 6.2 ± 9.8 | 1.5 ± 2.1 |
| 12 | 200:1 | 48.5 ± 11.7(6)[e] | 44.7 ± 18.0(3)[e] | 58.5 ± 12.9(4)[e] |
| | 100:1 | 33.3 ± 14.1 | 23.3 ± 11.7 | 45.5 ± 19.1 |
| | 50:1 | 12.7 ± 14.7 | 15.3 ± 11.7 | 34.0 ± 15.6 |
| | 25:1 | 5.2 ± 8.0 | 6.3 ± 10.8 | 20.3 ± 11.4 |

[a]E:T = Effector:Target.
[b]The 18 h $^{51}$Cr-release assay was performed on YAC-1 lymphoma cells as targets.
[c]s.d. = standard deviation.
[d]T.B. = tumour bearer.
[e]The numbers in parentheses indicate the number of tests performed. Each mouse was tested individually.
[f]The $^{125}$IUDR incorporation-inhibition assay was performed on B16-F10 melanoma cells as targets.

(1) Three month old mice bearing B16-F1 or B16-F10 tumours had cytostatic and NK activities similar to those of untreated control mice;

(2) Twelve month old mice bearing B16-F1 tumours also had NK and cytostatic activities similar to age matched controls;

(3) In contrast to these results 12 month old mice bearing the high metastasis B16-F10 tumours had a significant ($P<0.05$ in Student's *t*-test) decrease in their NK activity and a significant increase in their cytostatic activity. The increase in cytostasis (with B16-F10 cells as targets) can be interpreted either as an induced specific adaptive cellular activity against this syngeneic tumour or as a general increase in cytostasis.

Significant changes in cytotoxic and cytostatic activities were seen in 3 month old mice bearing s.c. B16 tumours or metastasis variants of this tumour in the abdominal region (Table II). In these mice, the NK activity markedly decreased and the cytostatic activity inereased.

### *NATA in 3 and 12 month old C57BL/6 mice inoculated with high or low B16 metastasis variants*

The sera of 3 or 12 month old mice inoculated with B16-F1 or with B16-F10 tumours or normal age matched controls were assayed for their ability to bind to formalin fixed B16 tumour cells. Tumour-bearer sera were assayed at various time intervals after tumour inoculation. The results of 2 experiments represented in Figure 5 show that sera from 3 month old B16-F10 inoculated but not from B16-F1 inoculated mice (28 or 35 days after inoculation of the tumour but still tumour free) had higher binding activity to B16 cells than sera either from age matched B16-F1 inoculated mice or non-inoculated controls. These results were reproducable as judged from the low standard deviation ranging from 1–10% of the mean. After reaching peak titres at 4 or 5 weeks after the inoculation of the B16-F10 tumour, the titres decreased as tumours continued to enlarge (45 days post inoculation).

We did not detect any difference in NATA titres against seemingly unrelated tumour target cells between tumour bearers and normal controls in either of the 2 age groups assayed (results not shown).

### Discussion

Three and 12 month old C57BL/6 mice differ in their ability to resist the development of tumours from a subthreshold number of syngeneic transplantable B16 melanoma cells. Some of the older mice resisted tumour growth whereas all the younger ones succumbed to their tumours. A similar phenomenon was seen when skin cancers were induced in mice by U.V. irradiation (Ebbessen & Kripke, 1982) and in mice exposed to certain chemical carcinogens (Stutman, 1975). In these cases tumour incidence, size and growth rate were lower in the older mice. In apparent contrast to

**Table II** NK and cytostatic activities mediated by splenocytes from normal mice and mice bearing B16-F1 and B16-F10 tumours growing s.c. in the abdominal region.

| | *NK activity (% $^{51}$Cr-release[b] (mean ± s.d.[c]))* | | | | | |
|---|---|---|---|---|---|---|
| | *Normal Control* (3)[e] | | *F1 T.B.*[d](3)[e] | | *F10 T.B.*[d](3)[e] | |
| *E:T*[a] *Ratio* | *Duration of the assay* 4h | 18h | 4h | 18h | 4h | 18h |
| 100:1 | 20.4±4.4 | 61.7±20 | 14.0±4.6 | 39.7±19.3 | 18.3±5.5 | 45.7± 9.0 |
| 50:1 | 14.0±3.0 | 47.6± 8 | 12.7±6.4 | 31.1±13.1 | 10.3±4.9 | 35.7± 5.0 |
| 25:1 | 11.7±3.5 | 31.3± 5 | 8.7±4.0 | 22.3±10.4 | 8.7±3.1 | 30.3± 2.1 |
| 12.5:1 | 11.0±6.1 | 23.3±11 | 6.7±4.7 | 19.0± 9.9 | 4.3±1.2 | 20.7± 8.0 |

| | *Cytostasis (% $^{125}$IUDR I-I[f] (Mean ± s.d.[c]))* | | |
|---|---|---|---|
| | *Normal Control (2)*[e] | *F1 T.B.*[d](2)[e] | *F10 T.B.*[d](2)[e] |
| 200:1 | 33.5±24.8 | 72.5±13.4 | 83.5± 9.2 |
| 100:1 | 15.5±19.1 | 60.8± 2.8 | 72.0±22.6 |
| 50:1 | 4.0± 5.7 | 35.5±17.7 | 47.0± 4.2 |
| 25:1 | 0±0 | 11.0± 8.5 | 17.5±12.0 |

Footnotes as in **Table I**.

**Figure 5** The binding of sera from normal (●), B16-F1 tumour inoculated (△) and B16-F10 tumour inoculated (▲) mice to B16 target cells. Sera from 3 month old mice (top) and sera from 12 month old mice (bottom) were utilized. Sera were drawn at different time intervals after tumour inoculation. Average values of 2 experiments are given. The reproducibility of the results of these experiments can be evaluated from the standard deviations given for 28 and 35 post inoculation days.

these results are observations that tumour growth is enhanced in old animals (Stutman, 1975). For example, certain transplanted lymphoma lines grow faster in old mice than in young mice (Haller *et al.*, 1977). It is therefore clear that no general rules can be formulated at present as to the effect of aging on the growth of induced or transplantable tumours.

It has already been proposed by Talmadge *et al.* (1980) that NK activity controls the outgrowth of inoculated B16 melanomas only if the cells comprising the inoculum are sensitive to NK-mediated lysis. The B16 cells utilized in our laboratory are resistant to lysis mediated by NK cells (Ehrlich *et al.*, 1981). It is not surprising therefore, that 12 month old mice, although having a decreased NK activity (as demonstrated in the short-term assays), are nonetheless able to resist the outgrowth of B16 cells at least as efficiently as 3 month old animals. It is thus safe to exclude a role for NK cells in this particular case, when dealing with NK-resistant local B16 tumours. On the other hand, our data do not exclude a role for NK cells in controlling metastasis formation, even in the B16 system. Indeed such a role has been demonstrated very convincingly (Hanna & Fidler, 1981; Hanna & Burton, 1981).

The results obtained in this study demonstrate also that older mice differ from younger ones in two additional respects: (1) Normal 12 month old mice have higher titres of NATA than younger animals against certain tumour targets; (2) Three month old mice inoculated with highly metastatic B16-F10 cells (but without palpable tumours) have anti B16 antibodies while similarly treated 12 month old mice have none. These phenomena could be responsible for the enhanced growth of B16 tumours in the younger mice. For instance, it is not unlikely that the increased NATA levels in 12 month old mice retard the proliferation of tumour cells in these mice. On the other hand, the high antibody titre to B16 cells in 3 month old B16 bearing mice possibly an adaptive response to antigens expressed on the tumour (Baniyash *et al.*, 1982) could facilitate the escape of tumour cells from destructive effectors and thus enhance tumour growth (Ehrlich & Witz, 1982).

We could not detect any alteration in any of the expressions of natural immunity (i.e. NK, cytostasis and NATA) during the tumour latency period. The decreased NK activity observed in some tumour bearers could thus be a result of the presence of a large tumour rather than its cause.

This investigation was supported by a grant from the Concern Foundation in conjunction with the Cohen-Appelbaum-Feldman Families Cancer Research Fund.

### References

BANIYASH, M., SMORODINSKY, N.I., YAAKUBOVICZ, M. & WITZ, I.P. (1982). Serologically detectable MHC and tumour-associated antigens on B16 melanoma variants and humoral immunity in mice bearing these tumours. *J. Immunol.*, **129**, 1318.

CHOW, D.A., WOLOSIN, L.B. & GREENBERG, A.H. (1981). Murine natural antitumour antibodies II The contribution of natural antibodies to tumour surveillance. *Int. J. Cancer*, **27**, 459.

CIKES, M., FREIBERG, S. & KLEIN, G. (1973). Progressive loss of H-2 antigens with concomitant increase of all surface antigen(s) determined by Moloney leukemia virus in cultured murine lymphomas. *J. Natl Cancer Inst.*, **50**, 347.

COLNAGHI, M.I., PIEROTTI, M.A., MENARD, S. & 3 others. (1979). Natural immune response in mice to tumour cells. In: *Current Trends in Tumour Immunology*. (Ed. Ferrone *et al.*), New York: Garland STPM Press, p. 3.

EBBESSEN, P. KRIPKE, M.L. (1982). Influence of age and anatomical site of ultraviolet carcinogenesis in BALB/c mice. *J. Natl Cancer Inst.*, **68**, 691.

EHRLICH, R., EFRATI, M., BAR EYAL, A. & 4 others. (1980). Natural cellular reactivities from mice bearing three types of primary tumours. *Int. J. Cancer*, **26**, 315.

EHRLICH, R., EFRATI, M. & WITZ, I.P. (1981). Some characteristics of natural cytostatic mouse splenocytes. *J. Immunol. Meth.*, **40**, 193.

EHRLICH, R., EFRATI, M. & WITZ, I.P. (1982). Further studies on the cytostatic activity mediated by murine splenocytes. In: *NK and other Natural Effector Cells*. (Ed. Herberman), New York: Academic Press, p. 201.

EHRLICH, R. & WITZ, I.P. (1982). Natural killer cells and naturally occurring antibodies as representatives of natural tumour immunity. *Pathobiol. Ann.*, **12**, 85.

FIDLER, I.J. (1973). Selection of successive tumour lines for metastasis. *Nature* (*New Biol.*), **242**, 148.

FISHER, G.A. & WELCH, A.D. (1957). Effect of citrovorum and peptones on mouse leukemia cells L5178Y in tissue culture. *Science*, **126**, 1018.

FRAKER, P.J. & SPECK, Jr. J.C. (1978). Tetrachloro 3a, 6a-diphenylglycoluril, a useful reagent for labelling proteins with $^{125}$I. *Biochem. Biophys. Res. Commun.*, **80**, 849.

HALLER, O., HANSSON, M., KIESSLING, R. & 1 other. (1977). Role of nonconventional natural killer cells in resistance to syngeneic tumour cells *in vivo*. *Nature*, **270**, 609.

HANNA, N. & BURTON, R.C. (1981). Definitive evidence that natural killer (NK) cells inhibit experimental tumour metastasis *in vivo*. *J. Immunol.*, **127**, 1754.

HANNA, N. & FIDLER, I.J. (1981). Relationship between metastatic potential and resistance to NK cell-mediated cytotoxicity in three murine tumour systems. *J. Natl Cancer Inst.*, **66**, 1183.

HAUGHTON, G. & WHITMORE, A.C. (1978). The effects of aging on immune function. In: *The Handbook of Cancer Immunology*. (Ed. Waters), New York: Garland STPM Press, vol. 1, p. 63.

HERBERMAN, R.B. & HOLDEN, H.T. (1978). Natural cell mediated immunity. *Adv. Cancer Res.*, **27**, 305.

HUANG, J.C.C., BERCZI, I., FROESE, G. & 2 others. (1975). A micro racioimmunoassay for antibodies to tumour associated antigens. *J. Natl Cancer Inst.*, **55**, 879.

KEAST, O. (1970). Immunosurveillance and cancer. *Lancet*, **ii**, 710.

KELLER, R. (1980). Regulatory capacities of mononuclear phagocytes with particular reference to natural immunity against tumours. In: *Natural Cell Mediated Immunity against Tumours*. (Ed. Herberman), New York: Academic Press, p. 1219.

KLEIN, E. & KLEIN, G. (1965). Antigenic properties of lymphomas induced by Moloney agent. *J. Natl Cancer Inst.*, **32**, 547.

KLEIN, G., EHLIN, B. & WITZ, I.P. (1979). Serological detection of a polyoma tumour associated membrane antigen. *Int. J. Cancer*, **23**, 683.

MAKINODAN, T. & KAY, M.M.B. (1980). Age influence on the immune system. *Adv. Immunol.*, **29**, 287.

MOAV, N., SMORODINSKY, N., BALIN, B.A. & 1 other. (1982). Monoclonal antibodies from mice bearing polyoma virus induced tumours. *Cancer Immunol. Immunother.*, **12**, 217.

NISONOFF, A., MARKUS, G. & WISSLER, F.C. (1961). Separation of univalent fragments of rabbit antibody by reduction of a single labile disulfide. *Nature*, **189**, 293.

PETO, R., PIKE, M.C., ARMITAGE, P. & 7 others. (1977). Design and analysis of randomised clinical trials requiring prolonged observation of each patient. *Br. J. Cancer*, **35**, 1.

SENDO, F., AOKI, T. & BOYSE, E.A. (1975). Natural occurrence of lymphocytes showing cytotoxic activity of BALB/c radiation-induced leukemia RLO 1 cells. *J. Natl Cancer Inst.*, **53**, 603.

STUTMAN, O. (1975). Immunodepression and malignancy. *Adv. Cancer Res.*, **22**, 261.

STUTMAN, O., FIGARELLA, E.F., PAIGE, C.J. & 1 other. (1980). Natural cytotoxic (NC) cells against solid tumours in mice: General characteristics and comparison to natural killer (NK) cells. In: *Natural Cell Mediated Immunity Against Tumours*. (Ed. Herberman), New York: Academic Press, p. 187.

TALMADGE, J.E., MEYERS, K., DRIERU, D.J. & 1 other. (1980). Role of NK cells in tumour growth and metastasis in beige mice. *Nature*, **284**, 622.

TYAN, M.L. (1981). Age related changes in marrow stem cells. In: *The Handbook of Cancer Immunology*. (Ed. Waters), New York: Garland STPM Press, vol. 6, p. 103.

WITZ, I.P., LEE, N. & KLEIN, G. (1976). Serologically detectable specific and cross-reactive antigens on the membrane of a polyoma virus-induced murine tumour. *Int. J. Cancer*, **18**, 243.

WITZ, I.P., YAAKUBOWICZ, M., GELERNTER, I., HOCHBERG, Y., ANAVI, R. & RAN, M. Studies on the level of natural antibodies reactive with various tumor cells during urethan carcinogenesis in BALB/c mice. *Immunobiology*. (In press).